# Nutritional Status Assessment Tools in Cardiovascular Patients

**DOI:** 10.3390/nu17162703

**Published:** 2025-08-20

**Authors:** Izabela Jarosz, Kamil Gorecki, Grzegorz Kalisz, Joanna Popiolek-Kalisz

**Affiliations:** 1 Department of Clinical Dietetics, Medical University of Lublin, ul. Chodzki 7, 20-093 Lublin, Poland; 2 Department of Bioanalytics, Medical University of Lublin, ul. Jaczewskiego 8b, 20-090 Lublin, Poland; 3 Department of Cardiology, Cardinal Wyszynski Hospital in Lublin, al. Krasnicka 100, 20-718 Lublin, Poland

**Keywords:** cardiovascular disease, malnutrition, nutritional status, GLIM, bioelectrical impedance analysis, phase angle, sarcopenic obesity

## Abstract

**Background:** Malnutrition is a prevalent but underrecognized condition in cardiovascular disease (CVD) patients, associated with adverse outcomes including longer hospitalizations, higher readmission rates, and increased mortality. Traditional measures such as body mass index (BMI) often fail to detect malnutrition, especially in patients with fluid retention, sarcopenia, or obesity. **Methods**: This review critically examines current tools used to assess nutritional status in CVD populations. Screening instruments such as Nutritional Risk Screening 2002 (NRS 2002), Mini Nutritional Assessment (MNA, MNA-SF), Malnutrition Universal Screening Tool (MUST), Subjective Global Assessment (SGA), and the Controlling Nutritional Status (CONUT) score are discussed, alongside diagnostic frameworks including the Global Leadership Initiative on Malnutrition (GLIM) criteria. The role of body composition assessment, particularly bioelectrical impedance analysis (BIA) and phase angle (PA), is also highlighted. **Results**: These tools differ in diagnostic performance and applicability, with many influenced by the pathophysiological features of CVD, such as inflammation, altered fluid balance, and pharmacotherapy. GLIM criteria provide a standardized two-step approach, combining phenotypic and etiologic factors, but require further validation in cardiology settings. **Conclusions**: A tailored, multimodal approach could be recommended: initial screening followed by confirmatory assessment using GLIM criteria and objective measures of muscle mass or cellular integrity. Clinicians should be aware of tool-specific limitations and interpret findings in the context of CVD-specific challenges.

## 1. Introduction

Malnutrition is one of the challenges in public health, contributing to poor nutritional status. It is a complex problem, often beginning before hospitalization and worsening during the hospital stay. This can lead to a decrease in muscle mass, hormonal dysfunctions, exacerbation of the underlying disease, and an increased incidence of other conditions. The decrease in muscle mass is also potentially associated with the wasting of the heart muscle, which is a poor prognostic factor in the course of cardiovascular diseases (CVDs) [1]. Cardiac cachexia is characterized by an involuntary loss of body weight, mainly lean body mass, and is strongly linked to adverse outcomes, including increased morbidity and mortality [2]. Its pathophysiology involves systemic inflammation, neurohormonal activation, metabolic dysregulation, and reduced nutrient intake, which promote skeletal and cardiac muscle catabolism [3]. Moreover, in patients with heart failure (HF), muscle mass loss is more associated with clinical outcomes than overall body mass loss alone [4]. It is worth noting that cardiac cachexia cannot be reversed by standard pharmacological therapy alone, highlighting the role of tailored nutritional interventions [5,6,7]. Its presence decreases exercise capacity and quality of life [4] and impacts mortality regardless of medications [2]. Thus, considering the prognostic role of cardiac muscle wasting, systematic nutritional screening and interventions in CVD populations are crucial in cachexia prevention.

CVD remains the leading cause of mortality with 20.5 million related deaths every year and a significant contributor to increasing healthcare costs globally [8,9]. It was reported that the burden is particularly high in low- and middle-income countries, where 80% of CVD-related deaths are reported, and where healthcare infrastructure may not be sufficient to address the growing demand [9]. Several factors, such as advanced age and the presence of comorbidities, are known to increase the risk and severity of CVD [8]. Non-modifiable factors include age, male sex, and genetic predisposition, while modifiable risk factors are hypertension, dyslipidemia, diabetes mellitus, obesity, and smoking [10]. They act synergistically and lead to atherosclerosis progression. Moreover, additional risk factors such as improper dietary patterns, physical inactivity, psychosocial stress, and excessive alcohol consumption increase CVD risk [10]. What is more, nutritional deficiencies have also been linked to negative CVD outcomes [11,12,13,14]. These risk factors not only increase the incidence of CVD but also pose a risk of malnutrition, leading to a potential vicious cycle that can potentially negatively affect both prognosis and quality of life.

Vitamin and mineral deficiencies in the course of malnutrition can worsen patient prognosis. Available evidence shows a relationship between low vitamin D blood levels and an increased risk of hypertension and coronary heart disease [15]. Vitamin D may inhibit the activity of the renin–angiotensin–aldosterone pathway, indicating that its deficiency may be one of the reasons for hypertension [11,16,17]. Cholecalciferol is also responsible for the proper condition of the vascular endothelium, and its deficiencies may cause endothelial damage, leading to the formation of atherosclerotic lesions [18,19]. Thiamine (vitamin B1) deficiency also is suggested to be a protective factor for the vascular endothelium [20]. Thiamine deficit also results in a decrease in ATP production in the heart muscle, resulting in its failure [20]. Hypomagnesemia can contribute to cardiac arrhythmias, which in turn increases mortality in patients with HF [18,21]. Vitamin B12 deficiency can lead to hyperhomocysteinemia [22], which is a risk factor for endothelial dysfunction, oxidative stress, and prothrombotic changes, linked to adverse cardiovascular outcomes, mainly stroke, in observational studies, but supplementation studies remain inconsistent [23,24,25,26]. Mendelian randomization analyses suggest the causal link for stroke, but not for coronary events [27].

Malnutrition affects up to 70% of patients with CVD, depending on the assessment method used, and is associated with a worsening clinical prognosis [28], including postoperative complications and deaths [29,30,31,32,33]. Malnutrition is particularly dangerous in the elderly, as the reduced physical exercise ability due to muscle wasting is an additional CVD risk factor [34]. Pharmacotherapy can cause reduced appetite, irritation of the gastrointestinal mucosa, impaired intestinal motility, and insufficient saliva production [18], which can additionally impact nutritional status. That is why the role of personalized nutritional interventions is emerging, as they may reduce hospital readmissions and improve survival, e.g., in HF patients [28]. On the other hand, there are currently no specific dietary guidelines tailored to HF patients that account for comorbidities and disease progression [4]. Malnutrition in HF is a multifactorial process involving neurohormonal activation, systemic inflammation, gastrointestinal congestion, and reduced appetite. Moreover, individuals suffering from obesity may paradoxically be undernourished, which is described as sarcopenic obesity, characterized by a decrease in muscle mass with an excessive level of adipose tissue [35]. As already mentioned, patients may also suffer from qualitative malnutrition, meaning a deficiency of vitamins and minerals despite a Body Mass Index (BMI) indicating obesity [36].

Therefore, this narrative review aims to provide a critical overview of validated tools for malnutrition screening and assessment in CVD populations, to guide clinicians beyond the limitations of BMI reliance.

## 2. Methods

This narrative review was conducted to provide a critical overview of tools used to assess nutritional status in CVD populations. A literature search was performed without period limitations in the PubMed, Scopus, and Web of Science databases. The search strategy combined terms related to nutritional status and CVD, including: “cardiovascular disease”, “heart failure”, “coronary artery disease”, “nutritional status”, “malnutrition”, “nutritional risk screening”, “NRS 2002”, “MUST”, “MNA”, “SGA”, “CONUT”, “GLIM”, “bioelectrical impedance analysis”, and “phase angle”. We included original research articles, systematic reviews, meta-analyses, and relevant narrative reviews published in English. Preference was given to studies conducted in adult populations and those specifically addressing the assessment of malnutrition or nutritional risk in CVD patients. Exclusion criteria included studies focusing on pediatric populations, animal models, or unrelated outcomes. Reference lists of key articles were also screened to identify additional relevant publications. Due to the range of the topic, the aim of this approach was not to provide an exhaustive systematic review but to synthesize the most clinically relevant evidence, highlighting current practices, methodological challenges, and research gaps in the assessment of nutritional status among CVD patients.

## 3. The Definition of Malnutrition

There are various definitions of malnutrition in the literature, developed by scientific societies and associations. According to European Society for Clinical Nutrition and Metabolism (ESPEN), malnutrition or undernutrition results from a lack of nutrients to the body, leading to weight loss and impairment of the mental and physical functions of the body [37]. Global Leadership Initiative on Malnutrition (GLIM) identifies two main mechanisms contributing to malnutrition: insufficient nutrient intake or absorption, and disease-related malnutrition. Both result in changes in body weight and impaired functioning of the body [38]. In contrast, the World Health Organization (WHO) defines malnutrition as a condition that includes both undernutrition and overnutrition, reflecting imbalances in energy and nutrient intake [39].

As indicated, there is no single definition of malnutrition. However, each of them mentions intake food disorders and changes in body composition. Moreover, GLIM distinguishes malnutrition in particular diseases. Given the clinical importance, establishing a unified definition and standardized diagnostic criteria for malnutrition would be clinically beneficial.

Malnutrition is often classified into two main categories: disease-related and intake-related [40]. Moreover, ESPEN categorized disease-related malnutrition into malnutrition related to inflammatory diseases and malnutrition related to non-inflammatory diseases [41]. A detailed description of each category is presented in Table 1.

Sarcopenia is a condition marked by the gradual weakening and deterioration of skeletal muscles. It typically involves both a measurable decline in muscle mass and a noticeable drop in strength or physical function [44]. In clinical practice, loss of muscle strength is often considered more relevant than muscle quantity alone when assessing the severity of this condition [45]. Findings from the SICA-HF study suggest that individuals with HF with reduced ejection fraction (HFrEF) are significantly more likely by a factor of 20 to develop sarcopenia compared to healthy peers of the same age [4]. A related already mentioned syndrome, known as sarcopenic obesity, occurs when excess body fat coexists with reduced muscle mass and diminished muscle function. This dual burden appears to be linked to chronic, low-grade inflammation and inactivity that often accompanies chronic illness [46]. Sarcopenic obesity is frequently observed in HF patients, particularly those with preserved ejection fraction (HFpEF), and is closely associated with limited exercise capacity and poor physical endurance [47,48,49].

The multifactorial etiology of malnutrition needs a structured and multidimensional approach to nutritional status assessment. ABCD is a widely accepted model based on four key domains: Anthropometric, Biochemical, Clinical, and Dietary [50]. Each component contributes information regarding the patient’s nutritional status, supporting both diagnostic accuracy and tailored intervention planning; however not all ABCD parts are equally useful in CVD settings. In the context of CVD, factors such as fluid overload, inflammation, sarcopenia, and polypharmacy can complicate the clinical overview. The potential parts representing ABCD are described in detail in the following paragraphs.

## 4. Anthropometric Measures

BMI is calculated as the ratio of body weight in kilograms to the square of height in meters and the obtained result categorizes individuals into underweight, normal body mass, overweight, and obesity. Its simplicity leads to wide use and it correlates with the prediction of an increased risk of metabolic diseases, but as mentioned its ability to predict the occurrence of metabolic diseases is limited [51]. Moreover, reliance on BMI alone may lead to an underestimation of the prevalence of malnutrition, as it does not differentiate between fat mass and lean body mass [52] and fails to detect occurrence of edema in the body [53,54]. Several studies have shown that BMI may inaccurately classify patients as well-nourished when more comprehensive assessment tools indicate malnutrition [51,55,56,57]. Moreover, waist circumference or waist-to-height ratio (WHtR) more accurately reflects visceral adiposity is a stronger predictor of CVD risk than BMI [58].

The mid-upper arm circumference (MUAC) is measured at the midpoint between the tip of the shoulder and the elbow on the non-dominant side of the body. It has proven to be a valid tool for detecting low muscle mass [59], whereas calf circumference (CC) reflects subcutaneous fat and bone mass. CC is also considered a surrogate marker of muscle mass for diagnosing sarcopenia [60]. Despite their ease of use and non-invasiveness, anthropometric measures such as CC and MUAC have notable limitations in patients with CVD, i.e., with these tools peripheral edema can lead to an overestimation of muscle mass, they also do not assess muscle quality [61].

Skinfold thickness (SFT) serves as a widely adopted simple anthropometric method for assessing nutritional status and detecting malnutrition across diverse populations. Lower SFT values correlate with higher undernutrition rates and align well with other assessment tools [62]. Notably, in a large cohort of lung cancer patients, low triceps skinfold thickness (TSFT), when combined with GLIM criteria, was significantly associated with a higher mortality risk [63]. Moreover TSFT added prognostic value beyond muscle mass and standard malnutrition assessments, underscoring its importance in identifying fat mass depletion in malnourished patients [63]. However, it is crucial to acknowledge that skinfold measurements are susceptible to significant measurement errors often due to observer technique or inter-observer differences [64]. The detailed comparison of anthropometric methods for malnutrition assessment in CVD patients was presented in Table 2.

## 5. Biochemical and Laboratory Markers and Inflammation Indicators

Among the biochemical indicators, blood levels of proteins such as albumin, prealbumin, and transferrin provide diagnostic value [65]. The role of visceral proteins in the context of malnutrition risk is described in the position of the American Society of Parenteral and Enteral Nutrition (ASPEN) [65]. Among the mechanisms that may contribute to the incomplete effectiveness of these laboratory tests in estimating the risk of abnormal nutritional status are: albumin displacement within the body, affecting its concentration in blood plasma, and reduction in albumin’s half-life, both of which occur as a result of inflammation in the body. The formation of edema due to low blood albumin levels is another factor [65]. In contrast, in another work, only the usefulness of prealbumin was analyzed [66]. Due to its short half-life, transthyretin concentration is a good indicator of malnutrition risk and the effectiveness of nutritional treatment, but for the same reason, it is not possible to identify patients suffering from malnutrition solely by testing its plasma level [67]. To analyze the results of laboratory tests concerning plasma visceral protein concentrations, the patient’s general condition should be considered. Data obtained from these tests relate to the prediction of treatment complications but do not necessarily correlate well with the assessment of malnutrition risk [67,68].

C-reactive protein (CRP) is an acute-phase inflammatory protein whose expression increases in response to inflammation, including in conditions such as rheumatoid arthritis, various cardiovascular diseases, and infections. It is widely used as a clinical biomarker of inflammation, and elevated serum CRP levels are a well-established independent predictor of CVD in asymptomatic individuals. Factors such as age, sex, smoking status, body weight, lipid profile, and blood pressure can influence CRP levels [67]. CRP levels may be a valuable component in evaluating the nutritional status of cardiovascular patients, as it can help differentiate between acute and chronic inflammatory conditions, supporting the accurate classification of malnutrition according to the GLIM criteria. In hospitalized patients, malnutrition is often not solely the result of inadequate nutrient intake, so the GLIM framework indicates disease-related malnutrition, which involves complex pathophysiological mechanisms. Both acute and chronic inflammation trigger the activation of the sympathetic nervous system, the immune response, and the hypothalamic–pituitary–adrenal (HPA) axis, which interact in response to physiological stress. Modulation of the HPA axis leads to the release of stress hormones such as cortisol and catecholamines, while suppressing hormones that regulate reproductive, thyroid, and other peripheral functions [42]. Therefore, the role of inflammation in the identification and treatment of malnutrition is essential and should not be overlooked.

The levels of total cholesterol, total lymphocyte count, and albumin are taken into account in calculating the Controlling Nutritional Status (CONUT) index. The result can be related to three point malnutrition risk categories: low, moderate risk, and high risk. However, the sensitivity of the CONUT index in detecting malnutrition is only 43%, as co-occurrence of diseases that may affect the values of cholesterol, albumin, and total lymphocyte concentrations [69]. Kinugasa et al. compared the MNA-SF, GNRI, and CONUT scores in patients with HF, and the CONUT index again showed lower effectiveness in assessing the nutritional status of patients, which was the results of the use of statins, which affect the total cholesterol level [70].

Coronary artery disease can be divided into acute coronary syndrome and chronic coronary syndrome. In the acute form, the limited or complete blockage of blood flow to the heart muscle results in acute myocardial ischemia which lead to resting chest pain [71]. In acute conditions, there is a high risk of developing malnutrition due to the sudden deterioration of physiological functions. In such cases, screening tools that rely heavily on retrospective information (e.g., weight loss or dietary intake over the past three to six months) may lack sensitivity and fail to identify patients at immediate nutritional risk [72]. On the other hand, the dominant symptom in chronic coronary syndromes is exertional angina, which may affect the daily functioning of patients, with even low levels of physical activity inducing pain and discomfort [73]. In these conditions, malnutrition can develop gradually and insidiously and both patients and physicians may fail to notice the slowly deteriorating nutritional status, leading to underdiagnosis. If malnutrition is not detected and treated in stable phases, the risk of poor outcomes increases significantly during acute deterioration. Therefore, continuous monitoring of nutritional status is essential at all stages of the CVD [74,75].

Biochemical parameters and inflammation markers provide additional insight into the potential etiology and severity of malnutrition in CVD patients. However, even though serum proteins have historically been used to assess nutritional status, their interpretation must consider the confounding impact fluid shifts, which are prevalent in CVD. Moreover, increased CRP levels, which is a marker of systemic inflammation, plays a dual role as it may not only reflect disease activity but also contribute directly to the pathogenesis of disease-related malnutrition through catabolic and anorexigenic pathways. That is why integrating laboratory markers with clinical and anthropometric data can support malnutrition classification, particularly in differentiation between starvation-related and inflammation-related malnutrition.

## 6. Body Composition Methods as Clinical Support for Malnutrition Diagnosis

Assessment methods for nutritional status also include body composition analysis using bioelectrical impedance analysis (BIA). This method is based on the passage of a low-intensity electrical current through the body’s soft tissues, measuring impedance [51,76,77]. Several parameters can be obtained, including fat-free mass (FFM), fat mass (FM), total body water (TBW), and phase angle (PA) [76]. PA based on resistance and reactance is particularly important in assessing nutritional status and predicting clinical outcomes. [78]. Cells with optimal energy levels, good metabolic condition and proper membrane structure and function, serve as good capacitators during current flow, causing a change in the phase of the alternating current relative to the source and high PA value. If a cell is unable to maintain proper cell membrane structure due to energy or nutrient deficiency, PA values will be reduced [79]. In CVD patients, particularly those with HF, BIA offers important advantages over traditional anthropometric tools like BMI [79,80]. BIA enables a precise assessment of tissue composition and allows for the monitoring of hydration status, which is especially relevant in patients receiving diuretic therapy or those undergoing decompensation. PA additionally serves as a prognostic marker of cellular integrity, with lower values associated with increased morbidity and mortality. Nevertheless, BIA is not without limitations. Measurements may be affected by the presence of metal implants, such as pacemakers or implantable cardioverter-defibrillators. Furthermore, inter-device variability and the lack of population-specific reference standards in CVD cohorts are further complicated interpretation. Therefore, although BIA is a useful adjunct to nutritional assessment, its results should always be interpreted within the broader clinical context.

Dual-energy X-ray absorptiometry (DXA) provides values for three major body composition components: FM, LBM, and bone mineral content (BMC). DXA uses low-dose X-rays, and through a detector interfaced with a computer imaging system, it enables comprehensive assessment of body composition. The radiation exposure from a whole-body DXA scan is less than 10% of that of a standard chest X-ray. DXA allows for the quantification of both whole-body and regional components. As such, it is considered an important technique for body composition assessment, with the ability to measure FM and LBM with accuracy comparable to computed tomography (CT) and magnetic resonance imaging (MRI) [81,82].

CT enables the assessment of visceral, intramuscular, subcutaneous, and skeletal muscle tissue, as well as fat infiltration into lean tissue. It is based on the emission and detection of X-rays. The key advantages of CT in body composition analysis include established reference thresholds, high image resolution, the ability to assess tissue quality, and high precision [83]. MRI is used to evaluate soft tissues by generating a magnetic field and emitting radio waves. It distinguishes between fat and water by exploiting the differences in magnetic resonance frequencies of hydrogen protons in fat and water. Importantly, MRI does not involve exposure to ionizing radiation [84]. However, both CT and MRI have limitations that restrict their routine clinical use. They are expensive, require trained specialists for image acquisition and interpretation, and may be contraindicated or poorly tolerated by some patients (e.g., due to claustrophobia or inability to remain still). Additionally, there is a lack of universally accepted cut-off values for defining “low muscle mass” based on CT or MRI data [81].

The potential advantage of body composition analysis in the assessment of malnutrition in CVD patients lies in its ability to differentiate between fat mass, lean body mass, and fluid compartments. This is a practical approach in conditions such as HF, where fluid overload is common. Moreover, FFM and PA serve as phenotypic markers of malnutrition and cellular integrity, as reflected in the GLIM criteria. Decreased PA was associated with adverse outcomes, including increased mortality and prolonged hospitalization in CVD patients. That is why body composition assessment with particularly BIA or DXA offers clinical data beyond BMI, improving malnutrition and sarcopenia identification in this complex population.

## 7. Dietary Assessment

The dietary component assessment is based on the evaluation of habitual food intake, recent changes in eating behavior, and potential barriers to adequate nutrition. In everyday clinical practice, particularly in CVD settings, detailed methods such as food frequency questionnaires, weighed food records, or 24 h dietary recalls are seldom used due to practical constraints [85]. Instead, a simplified approach is typically employed, based on short interviews conducted at admission or during follow-up visits. These include questions regarding recent changes in body weight, appetite, food quantity and quality, chewing or swallowing difficulties, and the presence of gastrointestinal symptoms such as nausea or early satiety. Despite its limitations, this short dietary interview can be still relevant for CVD patients. Disease-related factors, such as fatigue, dyspnea, or gastrointestinal congestion in HF, as well as polypharmacy and hospital food-related issues may contribute to inadequate intake. In addition, as already highlighted, qualitative malnutrition, defined as insufficient intake of essential nutrients despite adequate or excessive energy consumption, may occur in patients with obesity, a frequent phenotype in this population. Therefore, even a brief dietary assessment can provide clinically significant information on both caloric and micronutrient risk. Moreover, most validated screening tools, incorporate selected elements of dietary assessment, ensuring that this aspect is represented within standardized evaluation pathways [86,87].

## 8. Malnutrition Screening Scores

Nutritional Risk Screening 2002 (NRS 2002) is intended for the initial assessment of nutritional status in hospitals [86]. It consists of two main parts. The first part contains four questions concerning weight loss, reduced food intake, and diagnosed diseases. The second part includes categories for nutritional status and disease severity. If the patient is aged 70 years or older, they receive one additional point. The score of at least 3 points indicates the need for nutritional intervention [86]. NRS 2002 had been shown as a predictor of prolonged hospitalizations in patients undergoing invasive cardiac procedures including percutaneous coronary interventions and electrotherapy procedures [55,88,89].

The Malnutrition Universal Screening Tool (MUST) can be used both in a hospital and at home. It consists of three parts concerning BMI assessment, weight loss, and food intake assessment. A total score of 0 points indicates a low risk of malnutrition, 1 point indicates a medium risk, and a score of 2 or more points indicates a high risk of malnutrition, recommending nutritional intervention [90]. Brown et al. used MUST as part of the Malnutrition Pathway program implemented in primary care [91]. As a result of this initiative, patients received appropriate dietary interventions, which reduced the frequency of healthcare use and consequently lowered treatment costs [91]. Moreover, the MUST scale was the most useful preoperative nutritional assessment tool for predicting the risk of postoperative Activities of Daily Living decline in patients undergoing cardiovascular surgery [92]. These findings suggest that even a simple tool like MUST can effectively identify malnutrition and contribute to improved patient outcomes.

The Mini Nutritional Assessment (MNA) consists of 18 items. The first part is a screening section with 6 questions. If the total score is 11 or below, the patient proceeds to the next section to obtain the Malnutrition Indicator Score. The interpretation of the malnutrition index score is as follows: 24–30 points indicate normal nutritional status, 17–23.5 points indicate a risk of malnutrition, and below 17 points indicate malnutrition. The Mini Nutritional Assessment-Short Form (MNA-SF) includes only the 6 screening items from the full MNA and was developed to improve accessibility, particularly for older adults [87]. In a cross-sectional study conducted in a geriatric population, the MNA-SF identified fewer patients as malnourished or at risk of malnutrition compared to the full MNA and the agreement between the two versions was moderate [93]. An observational study of 121 outpatients with HF demonstrated that MNA, MUST, and SGA were useful for predicting malnutrition [94]. Moreover, MNA as a part of frailty screening tool was associated with reduced instrumental functional capacity and frailty led to increased length of hospital stay in elderly HF patients [95]. In summary, both the MNA and MNA-SF are useful in elderly patients and in CVD patients.

Screening of the nutritional status of hospitalized patients can also be carried out using the Subjective Global Assessment (SGA). It consists of three main components: an interview, a physical examination, and a subjective assessment of nutritional status. Based on the data obtained in the first two categories, the assessor can determine whether the patient is well-nourished, at risk of developing or worsening malnutrition during hospitalization, or if their nutritional status is already seriously impaired [96]. SGA was used in hemodialysis CVD patients; however, its role in CVD risk prediction was not established in this population [97].

The GLIM framework integrates phenotypic and etiologic criteria assessed after initial malnutrition risk assessment with recognized scores. Unintentional weight loss, low BMI, and low muscle mass are among the phenotypic criteria. The second group includes reduced intake or absorption, and diseases associated with inflammation. A diagnosis of malnutrition is confirmed when at least one phenotypic and one etiologic criterion are present. The severity of malnutrition is then graded based on the phenotypic criteria [38]. The GLIM criteria have faced scientific critique mainly for the phenotypic indicators, particularly unintentional weight loss and low BMI, due to alterations in body water distribution, which can compromise the accuracy of nutritional assessment in critically ill patients. It has also been noted that GLIM incorporates diagnostic components already used in other tools and, while highly sensitive, may lack specificity [98]. The MNA-SF scale was compared in the context of the GLIM criteria in a study involving 273 community-dwelling volunteers aged 60 years or older. All participants were screened using the MNA-SF and GLIM criteria. Malnutrition, as defined by GLIM, was diagnosed in 37.7% of participants. In contrast, the MNA-SF identified fewer cases, with under 10% classified as malnourished and 28.2% as at risk. The study’s authors suggested replacing the screening tool at the first step of the GLIM algorithm with clinical suspicion of malnutrition [99]. GLIM criteria are used as golden standard for abovementioned descriptive scores comparison in CVD populations [100]. The detailed comparison of used tools was summarized in Table 3.

Various malnutrition screening tools differ in structure, purpose, and suitability depending on the clinical context. NRS 2002 is intended for hospitalized patients and includes both nutritional status and disease severity. MUST is commonly used in both hospital and community settings due to its simplicity and applicability across a wide range of patients, including those in primary care. MNA and its short form MNA-SF are specifically designed for the elderly and offer a more detailed view of nutritional status, although MNA-SF may underestimate the number of at-risk patients compared to the full version. Most tools, such as NRS-2002, MUST, MNA, MNA-SF, SGA, and CONUT, were developed for general or geriatric populations and are not specifically tailored to the pathophysiological features of cardiovascular disease. Moreover, fluid overload, systemic inflammation, or sarcopenic obesity, often observed in CVD patients, may interfere with anthropometric and laboratory parameters, reducing the sensitivity or specificity of commonly used tools. For example, unintentional weight loss and BMI, which are central elements in most screening tools, may be impacted by fluctuations in hydration or masked by increased adiposity. Similarly, inflammatory conditions and pharmacological treatment may influence laboratory-based parameters such as cholesterol levels, limiting the reliability of tools like CONUT. Subjective tools, although holistic, may suffer from interobserver variability and limited reproducibility. Despite these limitations, nutritional screening remains a cornerstone of comprehensive cardiovascular care. In the work by Selcuk et al. the diagnostic accuracy of six commonly used malnutrition screening tools (MUST, MNA-SF, NRS 2002, Short Nutritional Assessment Questionnaire (SNAQ), Graz Malnutrition Screening (GMS)) in hospitalized older adults with CVD, using the GLIM criteria as the reference standard were evaluated [100]. When applied systematically, these tools support early identification of nutritional risk, which is a strong predictor of morbidity, hospital readmission, and mortality. That is why the tool selection should be guided by the clinical setting, patient characteristics, and available resources, with the understanding that no single instrument provides a complete picture of nutritional status in CVD [38,101]. To enhance diagnostic accuracy, screening results should ideally be followed by GLIM-based diagnostic assessment or body composition analysis.

## 9. Suggested Integrated Clinical Assessment Strategy

**Figure 1:**
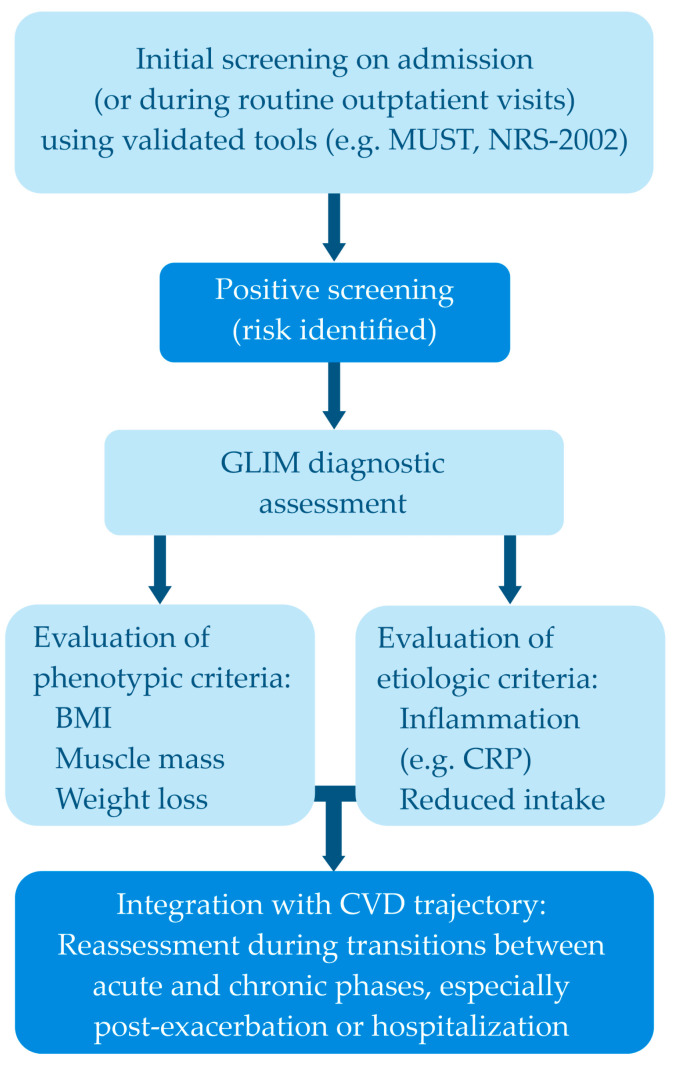
Suggested workflow (BMI—Body mass index, CVD—Cardiovascular diseases, CRP—C-reactive protein, GLIM—Global Leadership Initiative on Malnutrition, NRS 2002—Nutritional Risk Screening 2002, MUST—The Malnutrition Universal Screening Tool).

Given the high prevalence of unrecognized malnutrition in patients with CVD, a two-step approach combining screening tools with diagnostic confirmation is essential. While tools such as NRS 2002, MUST or MNA-SF are useful for early identification, they should be immediately followed by GLIM-based assessment in at-risk patients. The suggested workflow includes initial screening on admission (or during routine outpatient visits) using validated tools (e.g., MUST, NRS 2002); positive screening (i.e., risk identified) prompts full GLIM diagnostic assessment, integrated with CVD trajectory, i.e., reassessment during transitions between acute and chronic phases, especially post-exacerbation or hospitalization. This layered approach enhances sensitivity while maintaining clinical relevance and can help distinguish between disease-related and starvation-related malnutrition, which may influence intervention strategies. The suggested workflow was presented in Figure 1.

The implementation of nutritional assessment in cardiovascular settings must account for practical aspects such as time constraints, staff training, and documentation requirements. Most validated tools for screening and diagnosis, such as MUST, NRS-2002, MNA-SF, and the GLIM framework, are designed to be feasible in clinical conditions; however, their consistent application requires institutional commitment and interdisciplinary coordination. Feasibility largely depends on healthcare system organization and the recognition of nutritional risk as a clinical priority. In cardiology departments, routine nutritional screening may be overshadowed by disease-specific procedures, despite mounting evidence of its prognostic relevance. For instance, in Poland, the use of the NRS 2002 is mandated for all hospitalized patients according to national regulations. This presents a unique opportunity to systematically identify patients at nutritional risk, provided the tool is implemented accurately and followed by an appropriate clinical response. Training is essential for ensuring the reliability of assessment results. Nurses, dietitians, and physicians involved in the screening process should receive standardized education not only on tool administration but also on the interpretation of outcomes and the initiation of nutritional interventions. Variability in data quality often stems from inconsistent application or insufficient awareness of diagnostic thresholds, particularly in complex cases such as patients with fluid retention or sarcopenic obesity. Moreover, nutritional screening outcomes should be recorded upon hospital admission and reassessed during major clinical transitions, such as post-surgical recovery or discharge planning. Integration into electronic health records facilitates timely communication, supports continuity of care, and allows for quality monitoring and audits. In summary, while validated tools are available and mandated in many healthcare systems, their effectiveness relies on adequate training, institutional prioritization, and structured documentation. Embedding nutritional screening into cardiology practice requires a systems-level approach, linking administrative policy with clinical implementation.

Effective management of malnutrition in CVD needs an integrated, multidisciplinary approach. Identifying and mitigating nutritional risk requires the collaboration of various healthcare professionals. The input of clinical dietitians is particularly crucial, as they possess specialized knowledge in assessing nutritional status, planning personalized nutritional interventions, and monitoring their effectiveness, taking into account the specificities of cardiovascular diseases and ongoing therapies. Nurses play an invaluable role in daily patient care, conducting initial screenings, educating patients and their families, and supporting adherence to nutritional recommendations. Pharmacists can be involved in advising on potential interactions between nutritional supplements and cardiac medications. Physiotherapists can contribute significantly by assessing muscle function and supporting patients in maintaining physical activity, which is closely linked to nutritional status and muscle mass. Close collaboration among these professionals ensures comprehensive and holistic care for patients with CVD at risk of or suffering from malnutrition.

In addition to training for medical staff, educating patients and their families about the importance of proper nutrition in the context of cardiovascular diseases is fundamental to effective malnutrition management. Patients should be aware of how diet impacts their health status, how to recognize early signs of malnutrition, and what steps they can take to prevent or treat it. This education should include practical advice on composing meals rich in essential nutrients that are also consistent with dietary recommendations for cardiac patients. It should also emphasize the importance of regular monitoring of food intake and body weight and encourage open communication with the medical team about any difficulties related to nutrition. An informed patient, actively involved in their treatment process, is more likely to adhere to recommendations and achieve better health outcomes.

## 10. Limitations and Research Gaps

Although the awareness of malnutrition as a clinically relevant issue in CVD has increased, numerous limitations still hinder its effective identification and management in routine practice. Validated nutritional assessment tools such as MUST, NRS 2002, and MNA-SF are still rarely integrated into the standard workflow in cardiology departments or outpatient clinics. Nutritional status is often not systematically assessed, despite evidence that malnutrition can occur even in overweight or obese individuals with CVD [102,103].

Moreover, current diagnostic frameworks for malnutrition, such as the GLIM criteria, use generic cut-off points for indicators like BMI and unintentional weight loss. These cut-offs may not accurately reflect the physiological context of CVD, where factors such as fluid retention, sarcopenic obesity, or diuretic use can significantly affect body composition measurements. There is a need to validate and potentially adapt these criteria specifically for the CVD population [104]. Furthermore, studies assessing malnutrition in patients with HF, coronary artery disease, or atrial fibrillation often vary in design, population, tools used, and outcome measures. Some rely solely on anthropometric indicators, while others incorporate biochemical or imaging methods. These discrepancies limit data comparability and slow the development of robust, evidence-based guidelines [105]. Moreover, particularly in acute CVD, patients often experience rapid changes in hydration status and inflammatory responses. These fluctuations confound the accuracy of commonly used assessment tools. For example, hypoalbuminemia due to inflammation can mimic symptoms of malnutrition, while edema can lead to an overestimation of muscle mass or BMI. Failure to distinguish between these factors affects the diagnostic process [81].

## 11. Conclusions

Malnutrition contributes to muscle loss, impaired immune function, and reduced tolerance to medical therapies. In the context of CVD, it has been linked to increased morbidity, longer hospitalizations, readmissions, and higher mortality rates. Importantly, these outcomes are not exclusive to underweight patients; sarcopenia and unintentional weight loss may also occur in those with normal or elevated BMI, underscoring the need for comprehensive assessments. A two-step model, as recommended by the GLIM framework, offers a feasible and evidence-based approach. Initial screening using validated tools (e.g., MUST, NRS 2002) should be performed at hospital admission or during routine outpatient visits. Positive screening results should be followed by a diagnostic assessment incorporating phenotypic and etiologic criteria, including measurement of muscle mass and evaluation of inflammation (e.g., CRP).

Future research should aim to validate malnutrition diagnostic thresholds specifically in the CVD population, accounting for fluid shifts and inflammatory conditions. Additionally, protocols should be developed for the longitudinal monitoring of nutritional status across acute and chronic phases of CVD. There is also a need to explore how nutritional interventions, both preventive and therapeutic, can improve clinical outcomes and reduce healthcare costs. In summary, recognizing and managing malnutrition as an integral part of CVD care is not only clinically necessary but also a potentially cost-effective strategy to improve patient outcomes.

## Figures and Tables

**Table 1:** Comparison of types of malnutrition (based on literature data [41,42,43]).

Type of Malnutrition	Description	Clinical Effects
Disease-related malnutrition with inflammation	Malnutrition is associated with active disease with marked inflammation (e.g., malignancies, sepsis, COVID-19). Characterized by increased catabolism and weight loss despite energy intake.	- Nutritional therapy is less effective without proper inflammation control. - Pronounced muscle catabolism may occur even adequate calorie intake. - Direct impact on worsening prognosis and effectiveness of therapy for the underlying disease. - Increased risk of cachexia, mortality and prolonged hospitalization. - Requires multidirectional intervention (nutritional support, disease management and sometimes anti-inflammatory treatment).
Disease-related malnutrition without inflammation	Malnutrition is due to a chronic disease that interferes with the intake, digestion, or metabolism of nutrients but does not induce a significant inflammatory response (e.g., stroke, benign tumor, neurological disability).	- Nutritional therapy is often effective but requires concurrent treatment of the underlying disease. - Increased risk of functional complications (e.g., loss of strength and mobility). - Lack of inflammatory markers may delay diagnosis. - Moderate impact on the course of treatment of the underlying disease.
Starvation-related malnutrition	Malnutrition resulting from long-term energy and/or nutrient deficiencies. Typical in cases of starvation, anorexia, nutritional neglect.	- Usually, a good response to nutritional therapy once adequate intake is restored. - Catabolism is minimal or absent—slower loss of muscle mass. - Little or no effect on the metabolism of the underlying disease. - Risk of refeeding syndrome with early initiation of therapy.

**Table 2:** Comparison of anthropometric methods for malnutrition assessment in CVD patients.

Method	Description	Advantages	Limitations in Cardiac Patients	References
BMI	Ratio of weight (kg) to height^2^ (m^2^)	Simple, widely used; available in both specialist and outpatient settings; correlates with risk of metabolic diseases.	Cannot assess body composition, fat distribution, or edema; predictive ability for metabolic diseases limited; racial differences may affect interpretation; less accurate than waist circumference or WHtR for CVD risk prediction.	[51,52,53,54,55,56,57,58]
MUAC	Measured at midpoint between shoulder and elbow (non-dominant arm).	Valid tool for detecting low muscle mass compared to BIA and CT; easy and non-invasive.	Overestimation possible in presence of peripheral edema; does not assess muscle quality or cause of low lean mass.	[59,61]
CC	Reflects subcutaneous fat and bone mass; proxy for muscle mass due to large muscle volume in legs.	Useful surrogate for diagnosing sarcopenia; easy and low-cost.	Influenced by reduced mobility during illness; edema leads to overestimation of muscle mass; limited assessment of muscle quality.	[60,61]
SFT	Measures subcutaneous fat thickness; commonly TSFT (triceps).	Simple, cost-effective; reliable in resource-limited settings; correlates with undernutrition; adds prognostic value (e.g., lung cancer mortality risk).	Susceptible to high measurement error (imprecision and inaccuracy); observer technique and inter-observer variability limit reliability; may be less useful in fluid-retaining cardiac patients.	[62,63,64]

BMI—Body mass index, BIA—Bioelectrical impedance analysis, CC—Calf circumference, CT—Computer tomography imaging, CVD—Cardiovascular diseases, MUAC—mid-upper arm circumference, SFT—skinfold test, TSFT—triceps skinfold test, WHtR—waist-to-height ratio.

**Table 3:** Comparison of clinical malnutrition scores used in clinical practice.

Tool	Number of Items	Main Criteria	Advantages	Limitations
NRS 2002	2 parts (4 screening questions + nutritional status and disease severity)	Weight loss, reduced food intake, disease severity; +1 point if ≥70 years old	Widely used and recommended in hospitalized patients; incorporates disease severity	Less suited for community use; BMI- and weight-based criteria can be confounded by fluid overload; requires reliable body mass changes history
MUST	3 items (+ final risk classification)	BMI, weight loss, acute disease effect on intake	Simple and quick; used in hospitals and community; effective in primary care; reduces healthcare costs when integrated in care pathways	May underestimate malnutrition risk in fluid-overloaded or obese patients; relies on accurate weight history
MNA	18 items (screening + assessment)	Anthropometrics, dietary intake, lifestyle, medications, mobility, subjective perception	Designed for elderly; comprehensive; used for frailty and functional outcomes	Time-consuming; may overestimate normal status in overweight/obese elderly
MNA-SF	6 screening questions	Food intake, weight loss, mobility, stress/acute illness, neuropsychological problems, BMI	Quick and easy to use; recommended for geriatric screening; suitable for frailty assessments	Moderate agreement with full MNA; underestimates at-risk patients compared to MNA; limited in details
SGA	3 domains (history, exam, subjective judgment)	Weight change, dietary intake, gastrointestinal symptoms, functional capacity, muscle/fat loss, edema	Comprehensive; includes clinical judgment	Subjective; interobserver variability; predictive validity in CVD risk limited
CONUT	Based on 3 laboratory values	Albumin, total cholesterol, lymphocyte count	Objective, rapid, reproducible; based on routine labs	Influenced by inflammation, pharmacotherapy, and hydration; not specific for malnutrition in CVD
GLIM Criteria	2-step (screening + diagnosis)	Phenotypic: weight loss, low BMI, reduced muscle mass; Etiologic: reduced intake/absorption, disease burden/inflammation	Provides diagnostic framework; endorsed internationally; allows grading of severity	Criticized for limited sensitivity; BMI/weight loss may be confounded by hydration; overlaps with other tools; may require body composition methods

NRS 2002—Nutritional Risk Screening 2002, MUST—The Malnutrition Universal Screening Tool, MNA—The Mini Nutritional Assessment, MNA-SF—The Mini Nutritional Assessment—Short Form, SGA—Subjective Global Assessment, CONUT—CONtrolling NUTritional status, GLIM—Global Leadership Initiative on Malnutrition

## Data Availability

No new data was created in the course of the preparation of this manuscript.

## Abbreviations

The following abbreviations are used in this manuscript:

CVD	Cardiovascular diseases
HF	Heart Failure
BMI	Body Mass Index
ESPEN	European Society for Clinical Nutrition and Metabolism
WHO	World Health Organization
HFrEF	Heart failure and reduced ejection fraction
HFpEF	Heart failure with preserved ejection fraction
NRS-2002	Nutritional Risk Screening 2002
MUST	The Malnutrition Universal Screening Tool
MNA	The Mini Nutritional Assessment
MNA-SF	The Mini Nutritional Assessment-Short Form
SGA	Subjective Global Assessment
CONUT	CONtrolling NUTritional status
WHTR	waist-to-height ratio
MUAC	mid-upper arm circumference
CC	Calf Circumference
SFT	Directory of open access journals
BIA	bioelectrical impedance analysis
MR	Magnetic Resonanse Imaging
CT	Computer Tomography Imaging
FFM	Fat-free mass
FM	Fat mass
TBW	Total body water
PA	Phase angel
XC	reactance
DXA	Dual-energy X-ray absorptiometry
LBM	Lean Body Mass
BMC	bone mineral content
ASPEN	the American Society of Parenteral and Enteral Nutrition
CRP	C-reactive protein
HPA	hypothalamic-pituitary-adrena
GLIM	Global Leadership Initiative on Malnutrition

## References

[1] Malone A., Mogensen K.M. (2022). Key approaches to diagnosing malnutrition in adults. Nutr. Clin. Prac..

[2] Liu B., Wu X., Wang Y., Hu X. (2025). Association between cardiac cachexia and adverse outcomes in patients with heart failure: A meta-analysis of cohort studies. Heart.

[3] Saha S., Singh P.K., Roy P., Kakar S.S. (2022). Cardiac Cachexia: Unaddressed Aspect in Cancer Patients. Cells.

[4] Emami A., Saitoh M., Valentova M., Sandek A., Evertz R., Ebner N., Loncar G., Springer J., Doehner W., Lainscak M. (2018). Comparison of sarcopenia and cachexia in men with chronic heart failure: Results from the Studies Investigating Co-morbidities Aggravating Heart Failure (SICA-HF). Eur. J. Heart Fail..

[5] Rolfe M., Kamel A., Ahmed M.M., Kramer J. (2019). Pharmacological management of cardiac cachexia: A review of potential therapy options. Heart Fail. Rev..

[6] Prokopidis K., Isanejad M., Akpan A., Stefil M., Tajik B., Giannos P., Venturelli M., Sankaranarayanan R. (2022). Exercise and nutritional interventions on sarcopenia and frailty in heart failure: A narrative review of systematic reviews and meta-analyses. ESC Heart Fail..

[7] Kida K., Miyajima I., Suzuki N., Greenberg B.H., Akashi Y.J. (2023). Nutritional management of heart failure. J. Cardiol..

[8] Rashid M., Kwok C.S., Gale C.P., Doherty P., Olier I., Sperrin M., Kontopantelis E., Peat G., Mamas M.A. (2017). Impact of co-morbid burden on mortality in patients with coronary heart disease, heart failure, and cerebrovascular accident: A systematic review and meta-analysis. Eur. Heart J. Qual. Care Clin. Outcomes.

[9] Di Cesare M., Perel P., Taylor S., Kabudula C., Bixby H., Gaziano T.A., McGhie D.V., Mwangi J., Pervan B., Narula J. (2024). The Heart of the World. Glob. Heart.

[10] Visseren F.L.J., Mach F., Smulders Y.M., Carballo D., Koskinas K.C., Bäck M., Benetos A., Biffi A., Boavida J.-M., Capodanno D. (2021). 2021 ESC Guidelines on cardiovascular disease prevention in clinical practice. Eur. Heart J..

[11] Mozos I., Marginean O. (2015). Links between Vitamin D Deficiency and Cardiovascular Diseases. Biomed. Res. Int..

[12] DiNicolantonio J.J., Liu J., O’Keefe J.H. (2018). Magnesium for the prevention and treatment of cardiovascular disease. Open Heart.

[13] Serra M., Mollace R., Ritorto G., Ussia S., Altomare C., Tavernese A., Preianò M., Palma F., Muscoli C., Mollace V. (2025). A Systematic Review of Thiamine Supplementation in Improving Diabetes and Its Related Cardiovascular Dysfunction. Int. J. Mol. Sci..

[14] DiNicolantonio J.J., Liu J., O’Keefe J.H. (2018). Thiamine and Cardiovascular Disease: A Literature Review. Prog. Cardiovasc. Dis..

[15] Judd S., Tangpricha V. (2008). Vitamin D Deficiency and Risk for Cardiovascular Disease. Circulation.

[16] Pál É., Ungvári Z., Benyó Z., Várbíró S. (2023). Role of Vitamin D Deficiency in the Pathogenesis of Cardiovascular and Cerebrovascular Diseases. Nutrients.

[17] Li M. (2023). The role of vitamin D in chronic obstructive pulmonary disease with pulmonary hypertension. Pulm. Circ..

[18] Wleklik M., Uchmanowicz I., Jankowska-Polaå„Ska B., Andreae C., Regulska-Ilow B. (2018). The Role of Nutritional Status in Elderly Patients with Heart Failure. J. Nutr. Health Aging.

[19] Szymański F.M., Bomba-Opoń D.A., Łęgosz P., Głogowska-Szeląg J., Baran W., Szepietowski J.C., Kos-Kudła B., Filipiak K.J., Kozłowska-Wojciechowska M. (2015). Miejsce witaminy D w codziennej praktyce klinicznej—Interdyscyplinarne stanowisko ekspertów. Forum Med. Rodz..

[20] Eshak E.S., Arafa A.E. (2018). Thiamine deficiency and cardiovascular disorders. Nutr. Metab. Cardiovasc. Dis..

[21] Voultsos P.M.M., Bazmpani M.-A.M.M., Papanastasiou C.A.M.M., Papadopoulos C.E., Efthimiadis G., Karvounis H., Kalogeropoulos A.P., Karamitsos T.D. (2022). Magnesium Disorders and Prognosis in Heart Failure: A Systematic Review. Cardiol. Rev..

[22] Froese D.S., Fowler B., Baumgartner M.R. (2019). Vitamin B12, folate, and the methionine remethylation cycle-biochemistry, pathways, and regulation. J. Inherit. Metab. Dis..

[23] Undas A., Perła-Kaján J., Głowacki R. (2025). Homocysteine in adult patients with cardiovascular disease: Is it clinically relevant in 2025? A tribute to Hieronim Jakubowski (1946–2025). Pol. Arch. Intern. Med..

[24] Lentz S.R. (2005). Mechanisms of homocysteine-induced atherothrombosis. J. Thromb. Haemost..

[25] Li M., Ren R., Wang K., Wang S., Chow A., Yang A.K., Lu Y., Leo C. (2025). Effects of B Vitamins on Homocysteine Lowering and Thrombotic Risk Reduction-A Review of Randomized Controlled Trials Published Since January 1996. Nutrients.

[26] Polat E., Demir M.C., Kucukdemirci O. (2022). Investigation of Vitamin B12 Deficiency in Patients with Acute Coronary Syndrome and its Relationship with Gensini Score. Clin. Lab..

[27] Zhou F., He Y., Xie X., Guo N., Chen W., Zhao Y. (2025). Homocysteine and Multiple Health Outcomes: An Outcome-Wide Umbrella Review of Meta-analyses and Mendelian Randomization Studies. Adv. Nutr..

[28] Habaybeh D., De Moraes M.B., Slee A., Avgerinou C. (2021). Nutritional interventions for heart failure patients who are malnourished or at risk of malnutrition or cachexia: A systematic review and meta-analysis. Heart Fail. Rev..

[29] Popiolek-Kalisz J., Kalisz G., Zembala M. (2025). The Application of Bioelectrical Impedance Analysis Phase Angle in Cardiac Surgery. Nutrients.

[30] Martínez-Ortega A.J., Piñar-Gutiérrez A., Serrano-Aguayo P., González-Navarro I., Remón-Ruíz P.J., Pereira-Cunill J.L., García-Luna P.P. (2022). Perioperative Nutritional Support: A Review of Current Literature. Nutrients.

[31] Bae M.I., Shim J.-K., Lee H.S., Jeon S., Kwak Y.-L. (2025). Predictive value of postoperative prognostic nutritional index trajectory for mortality outcomes after off-pump coronary artery bypass surgery: A retrospective cohort study. Front. Nutr..

[32] Abe T., Inao T., Shingu Y., Yamada A., Takada S., Fukushima A., Oyama-Manabe N., Yokota I., Wakasa S., Kinugawa S. (2024). Associations of sarcopenia and malnutrition with 30-day in-hospital morbidity and mortality after cardiac surgery. Eur. J. Cardio-Thorac. Surg..

[33] Pavone N., Cammertoni F., Bruno P., Cutrone G., Chiariello G., Calabrese M., Grandinetti M., Nesta M., Marzetti E., Calvani R. (2024). Does a Poor Preoperative Nutritional Status Impact outcomes of Heart Valve Surgery?. J. Frailty Aging.

[34] Tonet E., Campana R., Caglioni S., Gibiino F., Fiorio A., Chiaranda G., Zagnoni S., Casella G., Campo G. (2021). Tools for the Assessment of the Malnutrition Status and Possible Interventions in Elderly with Cardiovascular Diseases. J. Clin. Med..

[35] Mirzai S., Carbone S., Batsis J.A., Kritchevsky S.B., Kitzman D.W., Shapiro M.D. (2024). Sarcopenic Obesity and Cardiovascular Disease: An Overlooked but High-Risk Syndrome. Curr. Obes. Rep..

[36] Hamed M., Zaghloul A., Halawani S.H., Fatani B.A., Alshareef B., Almalki A., Alsharif E., Alhadhrami S., Elmoneim H.M.A., ALhothaly Q.A. (2024). Prevalence of Overweight/Obesity Associated with Anemia Among Female Medical Students at Umm Al-Qura University in Makkah, Saudi Arabia: A Cross-Sectional Study. Cureus.

[37] Cederholm T., Barazzoni R., Austin P., Ballmer P., Biolo G., Bischoff S.C., Compher C., Correia I., Higashiguchi T., Holst M. (2017). ESPEN guidelines on definitions and terminology of clinical nutrition. Clin. Nutr..

[38] Cederholm T., Jensen G.L., Correia M.I.T.D., Gonzalez M.C., Fukushima R., Higashiguchi T., Baptista G., Barazzoni R., Blaauw R., Coats A.J. (2019). GLIM criteria for the diagnosis of malnutrition—A consensus report from the global clinical nutrition community. Clin. Nutr..

[39] World Health Organization Malnutrition—Questions and Answers. https://www.who.int/news-room/questions-and-answers/item/malnutrition.

[40] Jensen G.L., Mirtallo J., Compher C., Dhaliwal R., Forbes A., Grijalba R.F., Hardy G., Kondrup J., Labadarios D., Nyulasi I. (2010). Adult starvation and disease-related malnutrition: A proposal for etiology-based diagnosis in the clinical practice setting from the International Consensus Guideline Committee. JPEN J. Parenter. Enter. Nutr..

[41] Madini N., Vincenti A., Beretta A., Santero S., Viroli G., Cena H. (2024). Addressing Inflammaging and Disease-Related Malnutrition: Adequacy of Oral Nutritional Supplements in Clinical Care. Nutrients.

[42] Stumpf F., Keller B., Gressies C., Schuetz P. (2023). Inflammation and Nutrition: Friend or Foe?. Nutrients.

[43] Hegazi R., Miller A., Sauer A. (2024). Evolution of the diagnosis of malnutrition in adults: A primer for clinicians. Front. Nutr..

[44] Cruz-Jentoft A.J., Bahat G., Bauer J., Boirie Y., Bruyère O., Cederholm T., Cooper C., Landi F., Rolland Y., Sayer A.A. (2019). Sarcopenia: Revised European consensus on definition and diagnosis. Age Ageing.

[45] Damluji A.A., Alfaraidhy M., AlHajri N., Rohant N.N., Kumar M., Al Malouf C., Bahrainy S., Kwak M.J., Batchelor W.B., Forman D.E. (2023). Sarcopenia and Cardiovascular Diseases. Circulation.

[46] Cheng Y., Lin S., Cao Z., Yu R., Fan Y., Chen J. (2025). The role of chronic low-grade inflammation in the development of sarcopenia: Advances in molecular mechanisms. Int. Immunopharmacol..

[47] Wojzischke J., Van Wijngaarden J., van den Berg C., Yavuz A.C., Diekmann R., Luiking Y., Bauer J. (2020). Nutritional status and functionality in geriatric rehabilitation patients: A systematic review and meta-analysis. Eur. Geriatr. Med..

[48] Esteban-Fernández A., Villar-Taibo R., Alejo M., Arroyo D., Palomas J.L.B., Cachero M., Joaquin C., Bailón M.M., Pérez-Rivera J.Á., Romero-Vigara J.C. (2023). Diagnosis and Management of Malnutrition in Patients with Heart Failure. J. Clin. Med..

[49] Cereda E., Pedrolli C., Klersy C., Bonardi C., Quarleri L., Cappello S., Turri A., Rondanelli M., Caccialanza R. (2016). Nutritional status in older persons according to healthcare setting: A systematic review and meta-analysis of prevalence data using MNA ^®^. Clin. Nutr..

[50] Locks L.M., Parekh A., Newell K., Dauphinais M.R., Cintron C., Maloomian K., A Yu E., Finkelstein J.L., Mehta S., Sinha P. (2025). The ABCDs of Nutritional Assessment in Infectious Diseases Research. J. Infect. Dis..

[51] Gołacki J., Witczak K., Górecki K., Szafraniec-Porada A., Wronecki J., Porada D., Matyjaszek-Matuszek B. (2025). Bioimpedance Body Composition Analysis in Estimating Insulin Resistance in Women with Overweight and Obesity (LUCAS 1.1): A Retrospective Analysis. Clin. Diabetol..

[52] Barazzoni R., Jensen G.L., Correia M.I.T.D., Gonzalez M.C., Higashiguchi T., Shi H.P., Bischoff S.C., Boirie Y., Carrasco F., Cruz-Jentoft A. (2022). Guidance for assessment of the muscle mass phenotypic criterion for the Global Leadership Initiative on Malnutrition (GLIM) diagnosis of malnutrition. Clin. Nutr..

[53] Khanna D., Peltzer C., Kahar P., Parmar M.S. (2022). Body Mass Index (BMI): A Screening Tool Analysis. Cureus.

[54] Busetto L., Dicker D., Frühbeck G., Halford J.C.G., Sbraccia P., Yumuk V., Goossens G.H. (2024). A new framework for the diagnosis, staging and management of obesity in adults. Nat. Med..

[55] Popiolek-Kalisz J., Chrominski T., Szczasny M., Blaszczak P. (2024). Nutritional Status Predicts the Length of Stay and Mortality in Patients Undergoing Electrotherapy Procedures. Nutrients.

[56] Popiolek-Kalisz J., Blaszczak P. (2023). Nutritional Status of Coronary Artery Disease Patients-Preliminary Results. Int. J. Environ. Res. Public. Health.

[57] Guerrini A., Rovere L.D., Fernández-Jiménez R., Hardy-Añón C., Herola-Cobos C., Garcia-Olivares M., Fernández J.A., Sánchez F.H., Jiménez V.M., Aguilar I.V. (2025). The usefulness of the updated bioelectrical impedance vector analysis references for assessing malnutrition, sarcopenia and predicting mortality in hospitalized patients. Clin. Nutr..

[58] Powell-Wiley T.M., Poirier P., Burke L.E., Després J.-P., Gordon-Larsen P., Lavie C.J., Lear S.A., Ndumele C.E., Neeland I.J., Sanders P. (2021). Obesity and Cardiovascular Disease: A Scientific Statement From the American Heart Association. Circulation.

[59] Dijk D.G.-V., Weerink L.B., Milovanovic M., Haveman J.-W., Hemmer P.H., Dijkstra G., Lindeboom R., Campmans-Kuijpers M.J. (2021). Bioelectrical Impedance Analysis and Mid-Upper Arm Muscle Circumference Can Be Used to Detect Low Muscle Mass in Clinical Practice. Nutrients.

[60] Ceolin C., Acunto V., Simonato C., Cazzavillan S., Vergadoro M., Papa M.V., Trapella G.S., Sermasi R., Noale M., De Rui M. (2024). New Perspectives in the Association between Anthropometry and Mortality: The Role of Calf Circumference. J. Frailty Aging.

[61] Ishida Y., Maeda K., Nonogaki T., Shimizu A., Yamanaka Y., Matsuyama R., Kato R., Mori N. (2019). Impact of edema on length of calf circumference in older adults. Geriatr. Gerontol. Int..

[62] Ashtekar S.V., Padhyegurjar M.S., Padhyegurjar S.B., Powar J.D. (2022). Nutritional assessment with skinfold thickness and body- fat proportion in tribal and urban schoolchildren in Nashik district: A cross sectional study. J. Fam. Med. Prim. Care.

[63] Yin L., Fan Y., Lin X., Zhang L., Li N., Liu J., Guo J., Zhang M., He X., Liu L. (2022). Fat mass assessment using the triceps skinfold thickness enhances the prognostic value of the Global Leadership Initiative on Malnutrition criteria in patients with lung cancer. Br. J. Nutr..

[64] Ulijaszek S.J., Kerr D.A. (1999). Anthropometric measurement error and the assessment of nutritional status. Br. J. Nutr..

[65] Evans D.C., Corkins M.R., Malone A., Miller S., Mogensen K.M., Guenter P., Jensen G.L., the ASPEN Malnutrition Committee (2021). The Use of Visceral Proteins as Nutrition Markers: An ASPEN Position Paper. Nutr. Clin. Pract..

[66] Chiba T., Yokota J., Takahashi R., Sasaki K., Suzuki H. (2023). Prealbumin level is a predictor of activities of daily living at discharge in older patients with heart failure who became ADL-independent after hospitalization. Jpn. J. Compr. Rehabilitation Sci..

[67] Sproston N.R., Ashworth J.J. (2018). Role of C-Reactive Protein at Sites of Inflammation and Infection. Front. Immunol..

[68] Dellière S., Cynober L. (2017). Is transthyretin a good marker of nutritional status?. Clin. Nutr..

[69] Cabré M., Ferreiro C., Arus M., Roca M., Palomera E., Serra-Prat M. (2015). Evaluation of conut for clinical malnutrition detection and short-term prognostic assessment in hospitalized elderly people. J. Nutr. Health Aging.

[70] Kinugasa Y., Sota T., Kamitani H., Nakayama N., Nakamura K., Hirai M., Yanagihara K., Kato M., Ono T., Takahashi M. (2022). Diagnostic performance of nutritional indicators in patients with heart failure. ESC Heart Fail..

[71] Raat W., Nees L., Vaes B. (2025). Diagnostic accuracy of signs and symptoms in acute coronary syndrome and acute myocardial infarction: A diagnostic meta-analysis. Scand. J. Prim. Health Care.

[72] Lai A.R., Warrier M., Ng E.Z., Lin C., Chin Y.H., Kong G., Anand V.V., Lee E.C., Lai H., Ng H.W. (2023). Cardiovascular Outcomes in Acute Coronary Syndrome and Malnutrition. JACC Adv..

[73] Salinas G.L.A., Cepas-Guillén P., León A.M., Jiménez-Méndez C., Lozano-Vicario L., Martínez-Avial M., Díez-Villanueva P. (2024). The Impact of Geriatric Conditions in Elderly Patients with Coronary Heart Disease: A State-of-the-Art Review. J. Clin. Med..

[74] Hersberger L., Dietz A., Bürgler H., Bargetzi A., Bargetzi L., Kägi-Braun N., Tribolet P., Gomes F., Hoess C., Pavlicek V. (2021). Individualized Nutritional Support for Hospitalized Patients With Chronic Heart Failure. J. Am. Coll. Cardiol..

[75] Charkiewicz M., Wojszel Z.B., Kasiukiewicz A., Magnuszewski L., Wojszel A. (2023). Association of Chronic Heart Failure with Frailty, Malnutrition, and Sarcopenia Parameters in Older Patients—A Cross-Sectional Study in a Geriatric Ward. J. Clin. Med..

[76] Pawlak A., Ręka G., Olszewska A., Warchulińska J., Piecewicz-Szczęsna H. (2021). Methods of assessing body composition and anthropometric measurements—A review of the literature. J. Educ. Health Sport..

[77] Ward L.C., Brantlov S. (2023). Bioimpedance basics and phase angle fundamentals. Rev. Endocr. Metab. Disord..

[78] Cimmino F., Petrella L., Cavaliere G., Ambrosio K., Trinchese G., Monda V., D’angelo M., Di Giacomo C., Sacconi A., Messina G. (2023). A Bioelectrical Impedance Analysis in Adult Subjects: The Relationship between Phase Angle and Body Cell Mass. J. Funct. Morphol. Kinesiol..

[79] Popiolek-Kalisz J., Szczygiel K. (2023). Bioelectrical Impedance Analysis and Body Composition in Cardiovascular Diseases. Curr. Probl. Cardiol..

[80] Popiolek-Kalisz J., Kalisz G. (2025). Malnutrition assessed with phase angle and mortality risk in heart failure—Meta-analysis. Nutr. Metab. Cardiovasc. Dis..

[81] Marra M., Sammarco R., De Lorenzo A., Iellamo F., Siervo M., Pietrobelli A., Donini L.M., Santarpia L., Cataldi M., Pasanisi F. (2019). Assessment of Body Composition in Health and Disease Using Bioelectrical Impedance Analysis (BIA) and Dual Energy X-Ray Absorptiometry (DXA): A Critical Overview. Contrast Media Mol. Imaging.

[82] Messina C., Albano D., Gitto S., Tofanelli L., Bazzocchi A., Ulivieri F.M., Guglielmi G., Sconfienza L.M. (2020). Body composition with dual energy X-ray absorptiometry: From basics to new tools. Quant. Imaging Med. Surg..

[83] Ceniccola G.D., Castro M.G., Piovacari S.M.F., Horie L.M., Corrêa F.G., Barrere A.P.N., Toledo D.O. (2019). Current technologies in body composition assessment: Advantages and disadvantages. Nutrition.

[84] Kuriyan R. (2018). Body composition techniques. Indian. J. Med. Res..

[85] Shim J.-S., Oh K., Kim H.C. (2014). Dietary assessment methods in epidemiologic studies. Epidemiol. Health.

[86] Reber E., Gomes F., Vasiloglou M.F., Schuetz P., Stanga Z. (2019). Nutritional Risk Screening and Assessment. J. Clin. Med..

[87] Dent E., Hoogendijk E.O., Visvanathan R., Wright O.R.L. (2019). Malnutrition Screening and Assessment in Hospitalised Older People: A Review. J. Nutr. Health Aging.

[88] Popiolek-Kalisz J., Hollings M., Blaszczak P. (2025). Nutritional risk score predicts the length of stay in patients undergoing coronary angiography. Nutr Diet.

[89] Popiolek-Kalisz J., Błaszczak P. (2025). The impact of nutritional risk on the length of stay in patients undergoing percutaneous coronary interventions. Kardiol. Pol..

[90] Murphy J., Mayor A., Forde E. (2018). Identifying and treating older patients with malnutrition in primary care: The MUST screening tool. Br. J. Gen. Pract..

[91] Brown F., Fry G., Cawood A., Stratton R. (2020). Economic Impact of Implementing Malnutrition Screening and Nutritional Management in Older Adults in General Practice. J. Nutr. Health Aging.

[92] Oshima T., Tsutsumi R. (2025). The Malnutrition Universal Screening Tool (MUST) Predicts Postoperative Declines in Activities of Daily Living (ADL) in Patients Undergoing Cardiovascular Open-Heart Surgery. Nutrients.

[93] Schrader E., Grosch E., Bertsch T., Sieber C.C., Volkert D. (2016). Nutritional and functional status in geriatric day hospital patients–MNA short form versus full MNA. J. Nutr. Health Aging.

[94] Guillén R.L., Pla M.A., García A.M., de Miguel Á.D., Santana E.G., Sanchis S.M., Torres J.F.M. (2024). Nutritional Assessment in Outpatients with Heart Failure. Nutrients.

[95] Lisiak M., Jędrzejczyk M., Wleklik M., Lomper K., Czapla M., Uchmanowicz I. (2025). Nutritional risk, frailty and functional status in elderly heart failure patients. ESC Heart Fail..

[96] Jeejeebhoy K.N., Keller H., Gramlich L., Allard J.P., Laporte M., Duerksen D.R., Payette H., Bernier P., Vesnaver E., Davidson B. (2015). Nutritional assessment: Comparison of clinical assessment and objective variables for the prediction of length of hospital stay and readmission. Am. J. Clin. Nutr..

[97] Czaja-Stolc S., Potrykus M., Ruszkowski J., Dębska-Ślizień A., Małgorzewicz S. (2025). Nutritional Status, Uremic Toxins, and Metabo-Inflammatory Biomarkers as Predictors of Two-Year Cardiovascular Mortality in Dialysis Patients: A Prospective Study. Nutrients.

[98] Da Silva Passos L.B., De-Souza D.A. (2019). Some considerations about the GLIM criteria—A consensus report for the diagnosis of malnutrition. Clin. Nutr..

[99] Kaluźniak-Szymanowska A., Krzymińska-Siemaszko R., Lewandowicz M., Deskur-Śmielecka E., Stachnik K., Wieczorowska-Tobis K. (2021). Diagnostic Performance and Accuracy of the MNA-SF against GLIM Criteria in Community-Dwelling Older Adults from Poland. Nutrients.

[100] Selcuk K.T., Arslan S., Aydın A., Durmaz D. (2025). Which screening tool performs best in identifying malnutrition risk among hospitalized older adults with cardiovascular disease? A diagnostic accuracy study comparing six different screening tools with GLIM criteria. Eur. Geriatr. Med..

[101] Thanapholsart J., Khan E., Janwanishstaporn S., Thongma P., Naowapanich S., Pramyothin P., Chirakarnjanakorn S., Sethalao P., Tankumpuan T., Waldréus N. (2024). The assessment of reliability and validity of the Thai Versions of the Thirst Distress Scale for patients with Heart Failure and the Simplified Nutritional Appetite Questionnaire in heart failure patients. J. Res. Nurs..

[102] Bellanti F., Lo Buglio A., Quiete S., Vendemiale G. (2022). Malnutrition in Hospitalized Old Patients: Screening and Diagnosis, Clinical Outcomes, and Management. Nutrients.

[103] Cass A.R., Charlton K.E. (2022). Prevalence of hospital-acquired malnutrition and modifiable determinants of nutritional deterioration during inpatient admissions: A systematic review of the evidence. J. Human. Nutrition Diet..

[104] Driggin E., Cohen L.P., Gallagher D., Karmally W., Maddox T., Hummel S.L., Carbone S., Maurer M.S. (2022). Nutrition Assessment and Dietary Interventions in Heart Failure. J. Am. Coll. Cardiol..

[105] Miller J., Wells L., Nwulu U., Currow D., Johnson M.J., Skipworth R.J.E. (2018). Validated screening tools for the assessment of cachexia, sarcopenia, and malnutrition: A systematic review. Am. J. Clin. Nutr..

